# The Time Scale of Shallow Convective Self‐Aggregation in Large‐Eddy Simulations Is Sensitive to Numerics

**DOI:** 10.1029/2022MS003292

**Published:** 2023-01-04

**Authors:** Martin Janssens, Jordi Vilà‐Guerau de Arellano, Chiel C. van Heerwaarden, Bart J. H. van Stratum, Stephan R. de Roode, A. Pier Siebesma, Franziska Glassmeier

**Affiliations:** ^1^ Meteorology & Air Quality Department Wageningen University Wageningen The Netherlands; ^2^ Geoscience & Remote Sensing Department Delft University of Technology Delft The Netherlands; ^3^ Royal Netherlands Institute of Meteorology De Bilt The Netherlands

## Abstract

Numerical simulations of the tropical mesoscales often exhibit a self‐reinforcing feedback between cumulus convection and shallow circulations, which leads to the self‐aggregation of clouds into large clusters. We investigate whether this basic feedback can be adequately captured by large‐eddy simulations (LESs). To do so, we simulate the non‐precipitating, cumulus‐topped boundary layer of the canonical “BOMEX” case over a range of numerical settings in two models. Since the energetic convective scales underpinning the self‐aggregation are only slightly larger than typical LES grid spacings, aggregation timescales do not converge even at rather high resolutions (<100 m). Therefore, high resolutions or improved sub‐filter scale models may be required to faithfully represent certain forms of trade‐wind mesoscale cloud patterns and self‐aggregating deep convection in large‐eddy and cloud‐resolving models, and to understand their significance relative to other processes that organize the tropical mesoscales.

## Key Points

- In large‐eddy simulations, sub‐kilometer scale cumulus convection self‐organizes into mesoscale structures through shallow circulations
- The aggregation time‐scale does not converge with model resolution for typical discretization choices
- Numerical representations of the tropical mesoscales may require finer model resolutions than previously thought

## 1 Introduction

A striking feature of idealized simulations of the tropical atmosphere in radiative‐convective equilibrium (RCE) is the spontaneous aggregation of their column‐integrated moisture and convection into large clusters (Bretherton et al., 2005; Muller & Held, 2012). Many mechanisms have been proposed to explain this, including the collision and convective triggering of horizontally expanding and colliding cold pools of evaporated precipitation (Böing, 2016; Haerter, 2019; Tompkins, 2001) and gravity wave‐convection interactions (Yang, 2021). Yet, perhaps the strongest consensus is on the importance of shallow circulations (Muller et al., 2022; Shamekh et al., 2020), configured to transport moisture from dry to moist columns.

These circulations can be traced to differential, radiative cooling between moist regions, which trap outgoing longwave radiation in their moisture‐rich lower atmosphere and under high clouds, and dry regions, which more readily radiate their thermal energy to space (Muller & Held, 2012). Such heating anomalies give rise to ascent in moist columns and descent in dry columns, and may be framed as a moisture‐radiation instability (Beucler & Cronin, 2016; Emanuel et al., 2014) with negative moist gross stability (Bretherton et al., 2005; Raymond et al., 2009). However, the circulations may also be reinforced by turbulent mixing at cloud edges, which deposits moisture in the free troposphere and thus raises the livelihood and vigor of any subsequent convection; differential convection may then itself result in a net ascent of moist, convecting regions and descent in dry, non‐convecting regions (Grabowski & Moncrieff, 2004; Tompkins & Semie, 2017). Interactions between these radiative and convective feedbacks appear important, and their relative significance is debated (Beucler et al., 2018; Kuang, 2018).

Rooting deep convective self‐aggregation in shallow circulations implicitly underlines the importance of shallow convection in developing and maintaining them. Bretherton et al. (2005), Muller and Held (2012) make this connection explicit; they show that shallow convection in dry regions exports moist static energy, an appropriate energetic measure of the moisture, to moist, deep convective regions. If one removes cold‐pool feedbacks, the shallow circulation is even more tightly coupled to the effects of shallow, non‐precipitating convection. In such situations, self‐aggregation occurs also on smaller domains (Jeevanjee & Romps, 2013) and without requiring radiative feedbacks (Muller & Bony, 2015).

Interestingly, shallow cumulus convection under typical trade‐wind conditions also self‐organizes into clusters much larger than that of individual cumuli (e.g., Narenpitak et al., 2021). Bretherton and Blossey (2017), Janssens et al. (2022) attribute such aggregation to the convective feedback: Shallow circulations driven by anomalous latent heating in shallow cumulus transport moisture from dry to moist regions in the absence of any radiative or precipitating heterogeneity. If integrated over sufficiently long time periods, simulations of this mechanism aggregate enough moisture into their moist regions to transition into deep, organized convection (see also Vogel et al., 2016). These studies likely describe the confluence of shallow convective instability and the deep convective instabilities described by Jeevanjee and Romps (2013), Muller and Bony (2015), and grounds the latter in the former.

The paragraphs above serve to illustrate that an extensive body of work may rely rather strongly on how well the numerical models used to simulate convective self‐aggregation represent shallow convection. To remain tractable when running on domains of O(1,000) km, numerical simulations of self‐organization often employ rather coarse grid spacings (usually greater than 1 km). At such levels of discretization, the energetic scales of shallow convection—O(1) km—are at best barely resolved, and at worst parameterized. It is then natural to wonder whether under‐resolved shallow convection plays a role in explaining why convective self‐organization is so sensitive to numerical settings and parameterizations in cloud‐resolving simulations of deep convection (Muller & Held, 2012; Wing et al., 2020) and in large‐eddy simulations (LESs) of cold pool‐driven pattern formation in shallow convection (Seifert & Heus, 2013). This motivates us to ask the question: Can we consistently represent convective self‐aggregation in its most basic form—shallow, non‐precipitating cumulus convection—in LES?

Guided by this question, we revisit a classical case of non‐precipitating shallow cumulus convection and simulate it on a mesoscale domain in several numerical configurations (Section 2). We then summarize the feedback mechanism discussed by Bretherton and Blossey (2017), Janssens et al. (2022) that drives the self‐aggregation in these simulations (Section 3). Next, we demonstrate the multiscale nature of the feedback: Small, cumulus‐scale processes drive moisture variability at scales an order of magnitude larger (Section 4). This renders it sensitive to three choices that govern the effective resolution of finite‐volume‐based LES: grid spacing, advection scheme and unresolved turbulence model (Section 5). We discuss the implications of these findings for modeling studies that attempt to understand the relevance of shallow and deep convective self‐aggregation in nature, and for their potential parameterization in Section 6, before summarizing in Section 7.

## 2 Numerical Simulations

### 2.1 Case Study

Our study concerns a set of numerical experiments of the “undisturbed period” during the Barbados Oceanographic and Meteorological Experiment (BOMEX), as introduced to the LES modeling community by Siebesma and Cuijpers (1995). We concentrate on BOMEX because it represents the simplest imaginable setting of shallow cumulus convection, simulating only moist thermodynamics and boundary‐layer turbulence.

Our simulations run in the same configuration as reported by Siebesma et al. (2003). Three consequent assumptions deserve mention here. First, in lieu of representing spatial and temporal variability in (a) the large‐scale subsidence, (b) horizontal wind and (c) surface fluxes of heat and moisture, we parameterize larger‐scale forcings with profiles that vary only in height, and prescribe constant surface fluxes. Second, we do not locally calculate radiative heating rates, instead approximating them with a slab‐averaged cooling. Third, we explicitly ignore the formation and impact of precipitation. We will therefore suppress aggregation that is forced on our cloud‐field by (a) vertical motions of a scale larger than our domain, such as those imposed in the simulations conducted by Narenpitak et al. (2021) and observed by George et al. (2022), (b) radiative heterogeneity (Klinger et al., 2017) and (c) cold‐pool dynamics (e.g., Anurose et al., 2020; Lamaakel & Matheou, 2022; Seifert & Heus, 2013; Seifert et al., 2015), all of which appear important pathways to develop the mesoscale cumulus patterns observed in nature.

We justify the neglect of these processes by noting that they are not necessary for large, aggregated cumulus structures to develop (Bretherton & Blossey, 2017). Instead, they accelerate and modulate an internal mechanism that also occurs without them. This feedback is intrinsic to moist, shallow convection (Janssens et al., 2022), and its sensitivity to resolution is most clearly exposed by only studying this aspect. Yet, we will return briefly to the consequences of these assumptions in Section 6.

### 2.2 Numerical Model

We perform simulations with two models: The Dutch Atmospheric Large Eddy Simulaton (DALES, Heus et al., 2010; Ouwersloot et al., 2017) model and MicroHH (Van Heerwaarden et al., 2017). Both models attain a numerical representation of the atmospheric state on a staggered grid by solving filtered, finite difference approximations of the conservation equations of mass, momentum, and scalars in the anelastic approximation:

(1) $$\frac{\partial }{\partial x_{j}}\left(\rho_{0}u_{j}\right)=0$$

(2) $$\frac{\partial u_{i}}{\partial t}=-\frac{1}{\rho_{0}}\frac{\partial }{\partial x_{j}}\left(\rho_{0}u_{i}u_{j}\right)-\frac{\partial \pi^{\prime }}{\partial x_{i}}+\frac{g}{\bar{\theta_{v}}}\left(\theta_{v}-\bar{\theta_{v}}\right)\delta_{i3}-\frac{\partial \tau_{ij}}{\partial x_{j}}+S_{u_{i}}$$

(3) $$\frac{\partial \chi_{i}}{\partial t}=-\frac{1}{\rho_{0}}\frac{\partial }{\partial x_{j}}\left(\rho_{0}u_{j}\chi_{i}\right)-\frac{\partial R_{u_{j},\chi_{i}}}{\partial x_{j}}+S_{\chi_{i}},$$

In these equations, *u*_*i*_ ∈ {*u*, *v*, *w*} are the three (grid‐filtered) components of velocity, *χ*_*i*_ ∈ {*θ*_*l*_, *q*_*t*_} is a generic scalar whose set contains at least the total specific humidity *q*_*t*_ and liquid‐water potential temperature, approximated as

(4) $$\theta_{l}\approx \theta -\frac{L_{v}}{c_{p}\Pi }q_{l}.$$

where *θ* is the (dry) potential temperature, *L*_*v*_ is the latent heat of vaporization, *c*_*p*_ is the specific heat of dry air at constant pressure, *q*_*l*_ is the liquid water specific humidity and

(5) $$\Pi ={\left(\frac{p}{p_{0}}\right)}^{\frac{R_{d}}{c_{p}}}$$

is the Exner function, where *R*_*d*_ is the gas constant of dry air and *p* is the reference pressure profile. The corresponding reference density is *ρ*_0_, π′ are fluctuations of modified pressure around *p*, *g* is gravitational acceleration, *θ*_*v*_ is the virtual potential temperature whose slab‐mean is represented by an overbar, $S_{u_{i}}$ and $S_{\chi_{i}}$ denote momentum and scalar sources, and *τ*_*ij*_ and $R_{u_{j},\chi_{i}}$ are the residual fluxes of momentum and scalars that result from filtering the equations (the sub‐filter scale (SFS) fluxes, sometimes also referred to as sub‐grid scale fluxes). These fluxes are approximated with a traditional eddy viscosity model, which explicitly assumes the filtering to take place at a scale where diffusion of the resolved flow approximates the net dissipation of homogeneous, isotropic turbulence; it must be significantly smaller than the energy‐containing scales of the simulation:

(6) $$\tau_{ij}\approx -K_{m}\left(\frac{\partial u_{i}}{\partial x_{j}}+\frac{\partial u_{j}}{\partial x_{i}}\right)$$

(7) $$R_{u_{j},\chi_{i}}\approx -K_{h}\frac{\partial \chi_{i}}{\partial x_{j}}$$

These approximations introduce modeling errors which can be expected to influence the large, resolved scales when their requirements are not met.

The main differences between DALES and MicroHH reside in their model for the eddy diffusivities *K*_*m*_ and *K*_*h*_: DALES uses a one‐equation closure for the turbulent kinetic energy *e* (Deardorff, 1973) subject to Deardorff (1980)'s stability correction; MicroHH employs a stability‐corrected Lilly‐Smagorinsky model (Lilly, 1968). Both models estimate *K*_*m*_ and *K*_*h*_ through a mixing length *λ* associated with the grid‐scale filter:

(8) $$\lambda =f\left(\Delta \right),$$

(9) $$\Delta ={\left(\Delta x\Delta y\Delta z\right)}^{\frac{1}{3}},$$

where *f* subsumes the stability correction, which diminishes the eddy diffusivities in stably stratified grid cells, and where Δ assumes the grid spacing is isotropic, which is an assumption we will violate. Note that Δ also sets the discretization error in the model's spatial gradients for a finite difference scheme of a given order; these errors will interact non‐trivially with the modeling error made by the approximations above.

### 2.3 Experiments

We base our analysis on 10 simulations of BOMEX that vary in their choice of computational grid, advection scheme and SFS model (Table 1). To support mesoscale fluctuations with little influence from the finite domain size, the cases are run on domains with horizontal length *L* = 102.4 km, a height of 10 km, for 36 hr. All simulations have a vertical grid spacing Δ*z* = 40 m up to 6 km, stretched by 1.7% per level above this height. To investigate how the development of mesoscale fluctuations is sensitive to numerics, we vary the horizontal grid spacing $\Delta x=\Delta y\in \left[50,100,200\right]$ m. At their coarsest spacing, our grid cells attain rather high aspect ratios. Although such anisotropic grids are commonly used in large‐domain LES of shallow cumulus convection (e.g., Bretherton & Blossey, 2017; Janssens et al., 2022; Klinger et al., 2017; Vogel et al., 2016), the isotropic filter length scale *λ* consequently overestimates the vertical length scale required from the SFS model, and underestimates the horizontal length scale (de Roode et al., 2022). As will become clear in Section 5, we will be particularly concerned with this underestimation. Therefore, we also run the DALES simulations at Δ*x* = 200 m and Δ*z* = 40 m with Δ manually set to 200 m.

**Table 1:** Differences in Numerical Configurations of BOMEX Simulations

Abbreviation	Model	Δ*x*	SFS model	Adv. scheme	Δ	Hours analyzed
D1*	DALES	200	*e*	O(2) a2	117	6–17
D2	DALES	200	*e*	O(5) a5	117	6–36
D3*	DALES	200	*e*	O(2) a2	200, fiso	6–24
D4	DALES	100	*e*	O(2) a2	73.7	6–22
D5*	DALES	100	*e*	O(5) a5	73.7	6–36
D6*	DALES	100	*e*	O(5) a2	73.7, nocorr	6–24
D7	DALES	50	*e*	O(2) a2	46.4	6–32
M1	MicroHH	200	SL	O(2) a2	117	6–12
M2	MicroHH	100	SL	O(2) a2	73.7	6–36
M3	MicroHH	50	SL	O(2) a2	46.4	6–36

*Note*. Advection schemes are either O(2) central differences (a2, effective resolution of order 3Δ*x*), or the O(5) scheme by Wicker and Skamarock (2002) (a5 effective resolution of order 6Δ*x*). “fiso” refers to coarsening the filter as if it were isotropically increasing with the horizontal grid spacing, while “nocorr” denotes a run with Deardorff (1980)'s stability correction turned off. Simulations marked with * are additionally rerun starting from simulation D4 at 12 hr for the analysis performed in Section 5. *e* refers to the one‐equation turbulence kinetic energy sub‐filter scale model (Deardorff, 1973); SL refers to the smagorinsky‐lilly Model (Lilly, 1968).

All cases that vary Δ*x* are run with a variance‐preserving, second order central difference scheme to represent advective transfer. The coarsest two DALES simulations (D2 and D5) are additionally repeated using a fifth order, nearly monotonic scheme (Wicker & Skamarock, 2002) for horizontal advection (vertical advection is always computed with the second order scheme). The fifth‐order scheme is rather diffusive, consequently dampens the (co)variance contained in the smallest, resolved scales of the simulations we run (Heinze et al., 2015), and has an effective resolution of 6Δ*x*—commensurate with the five grid‐point stencil it requires (Bryan et al., 2003). As we shall see, these properties have significant consequences. Finally, we test the effects of the stability correction on *λ* by running a single simulation where it is turned off.

We focus on the period after an unaggregated cumulus layer has developed, but before any characteristic moisture length scales approach the domain size of our simulations. This eliminates model spinup and finite‐domain constraints posed by our doubly‐periodic boundary conditions respectively. The resulting analysis times for each simulation are reported in Table 1.

## 3 Conceptual Model for Self‐Aggregation

We will study the numerical sensitivity of the shallow convective self‐aggregation using the conceptual model described by Janssens et al. (2022), which is a closed‐form version of the theory introduced by Bretherton and Blossey (2017). The model is briefly summarized in this section; readers looking for elaboration are encouraged to explore the above manuscripts.

### 3.1 Definitions

In the following, self‐aggregation of the convection in our simulations will be interpreted as growth in mesoscale fluctuations of vertically integrated moisture. To make this more precise, let us define mesoscale fluctuations in a generic scalar *χ* by partitioning it into its slab‐average $\bar{\chi }$ and remaining fluctuation *χ*′, before scale‐separating *χ*′ into a mesoscale component $\chi_{m}^{\prime }$ and sub‐mesoscale component $\chi_{s}^{\prime }$:

(10) $$\chi =\bar{\chi }+\chi^{\prime }=\bar{\chi }+\chi_{m}^{\prime }+\chi_{s}^{\prime }.$$

$\chi_{m}^{\prime }$ is defined with a spectral low‐pass filter at 12.5 km, that is, fluctuations larger than this scale are considered mesoscale fluctuations.

In our framework, self‐aggregation is associated with the development of coherent mesoscale regions that are moist and convecting, where $q_{t_{m}}^{\prime }>0$, and dry, non‐convecting regions, where $q_{t_{m}}^{\prime }<0$. To identify these regions in our simulations, we use the density‐weighted vertical integral

(11) $$\langle \chi \rangle =\int_{0}^{z_{\infty }}\rho_{0}\chi dz,$$

where *z*_*∞*_ = 10 km, yielding the column‐integrated moisture 〈*q*_*t*_〉. In the following, positions where $\langle q_{t_{m}}^{\prime }\rangle >0$ are referred to as moist, mesoscale regions; locations where $\langle q_{t_{m}}^{\prime }\rangle <0$ are dry mesoscale regions.

With these definitions, we formulate a budget for $\chi_{m}^{\prime }$ by subtracting the slab‐average of Equation 3 from itself, mesoscale‐filtering the result, and rewriting several terms:

(12) $$\frac{\partial \chi_{m}^{\prime }}{\partial t}=\underset{\text{Grad.}\;\text{prod.}}{\underset{︸}{-w_{m}^{\prime }\Gamma_{\chi }}}\underset{\text{Horizontal}\;\text{transport}}{\underset{︸}{-\frac{\partial }{\partial x_{j_{h}}}{\left(u_{j_{h}}\chi^{\prime }\right)}_{m}}}\underset{\text{Vertical}\;\text{transport}}{\underset{︸}{-\frac{1}{\rho_{0}}\frac{\partial }{\partial z}\left(\rho_{0}F_{\chi_{m}^{\prime }}\right)}}\underset{\text{Subsidence}}{\underset{︸}{-\bar{w_{ls}}\frac{\partial \chi_{m}^{\prime }}{\partial z}}}\underset{\text{SFS}\;\text{diffusion}}{\underset{︸}{+\frac{\partial }{\partial x_{j}}\left(R_{{u_{j},\chi^{\prime }}_{m}}\right)}}\underset{\text{Source}}{\underset{︸}{+S_{\chi_{m}}^{\prime }}}$$

In this relation, the slab‐averaged vertical gradient $\partial \bar{\chi }/\partial z=\Gamma_{\chi }$, while $F_{\chi_{m}^{\prime }}$ is the anomalous mesoscale vertical flux of *χ*′ around the slab average

(13) $$F_{\chi_{m}^{\prime }}={\left(w^{\prime }\chi^{\prime }\right)}_{m}-\bar{w^{\prime }\chi^{\prime }}.$$

The conceptual model requires Equation 12 to be posed for measures of moisture and heat. To remain consistent with Bretherton and Blossey (2017), Janssens et al. (2022), we will use *q*_*t*_ as our moisture variable, and liquid‐water virtual potential temperature, defined as

(14) $$\theta_{lv}=\theta_{l}+0.608\bar{\theta_{l}}q_{t}\equiv \theta_{v}-7\bar{\theta_{l}}q_{l},$$

as our heat variable (e.g., B. Stevens, 2007). Both *q*_*t*_ and *θ*_*lv*_ are conserved under non‐precipitating shallow cumulus convection. Hence, in the absence of radiative heterogeneity, we immediately recognize that $S_{\chi_{m}}^{\prime }=0$. We will additionally assume that the direct effects of horizontal transport, subsidence and SFS diffusion on the $\chi_{m}^{\prime }$ budget are small (Figures S1 and S2 in Supporting Information S1, Janssens et al., 2022).

### 3.2 Model

**Figure 1:**
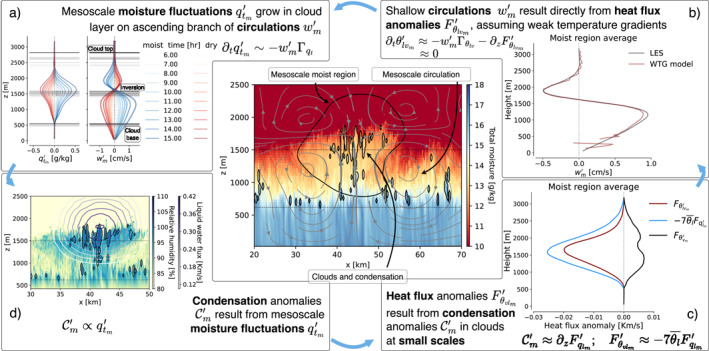
Overview of the circulation‐driven self‐aggregation mechanism in simulation D1 after 16 hr. Central panel: Example *x*‐*z* cross‐section depicting clouds (small, jagged black contours), which form favorably on a moist, mesoscale region (colored contours; large, smooth, black contour), in turn formed by a mesoscale circulation (streamlines). Horizontal lines indicate the cloud and inversion bases. (a) Vertical profiles of $q_{t_{m}}^{\prime }$ and $w_{m}^{\prime }$, averaged over moist (blue) and dry (red) mesoscale regions, evolving in time (increasing opacity). (b) Weak Temperature Gradient approximation Equation 17 (maroon) of $w_{m}^{\prime }$ compared to LES‐diagnosed ground‐truth (black). (c) Mesoscale heat flux anomaly $F_{{\theta_{lv}^{\prime }}_{m}}$ (maroon, using Equation 13), its liquid water flux approximation (blue, using Equation 20) and the buoyancy flux anomaly $F_{\theta_{v_{m}}^{\prime }}$, which is comparatively small. (d) As in central panel, but colored by relative humidity and overlaid by contours of $7\bar{\theta_{l}}{\left(w^{\prime }q_{l}^{\prime }\right)}_{m}$.

The main features of the conceptual model are captured in Figure 1. Its central panel shows a vertical cross‐section of simulation D1 after 16 hr of simulation time, colored by *q*_*t*_. Clouds are drawn on top of the *q*_*t*_ field as small, black contour lines. They form preferentially on an anomalously moist, mesoscale patch in the cloud layer (smooth, black contour line, delineating the boundary where $q_{t_{m}}^{\prime }=0$); convection and clouds have self‐aggregated into mesoscale structures in this panel.

To explain why, we begin at Figure 1a, which shows a progressing contrast in $q_{t_{m}}^{\prime }$ between moist (blue) and dry (red) regions near the inversion base. Upon vertically integrating Equation 12, the resulting increase in $\langle q_{t_{m}}^{\prime }\rangle$ can primarily be attributed to the “gradient production” term (Janssens et al., 2022, Figure 8) (Bretherton & Blossey, 2017, Figure 13), that is,

(15) $$\frac{\partial \langle q_{t_{m}}^{\prime }\rangle }{\partial t}\approx -\langle w_{m}^{\prime }\Gamma_{q_{t}}\rangle .$$

This term expresses transport along the mean, negative moisture gradient with mesoscale vertical velocity anomalies $w_{m}^{\prime }$, which in Figure 1a grow increasingly positive in the moist cloud layer, and increasingly negative in the dry cloud layer. $w_{m}^{\prime }$ embodies the ascending and descending branches of a shallow circulation (drawn as in‐plane streamlines in the central panel of Figure 1), which converges in the moist regions' subcloud layer, transports mixed‐layer moisture into the corresponding, moist cloud layer, and diverges near the trade‐inversion base into dry regions, where it subsides.

The shallow circulations $\left(w_{m}^{\prime }\right)$ may be understood as a direct result from heat flux differences between moist and dry mesoscale regions. To show this, consider Figure 1b. It plots $w_{m}^{\prime }$, averaged over the moist, mesoscale regions as (a) diagnosed by the LES model, and (b) as predicted by reducing Equation 12 for *θ*_*lv*_ to a diagnostic relation:

(16) $$\frac{\partial \theta_{lv_{m}}^{\prime }}{\partial t}\approx -w_{m}^{\prime }\Gamma_{\theta_{lv}}-\frac{1}{\rho_{0}}\frac{\partial }{\partial z}\left(\rho_{0}F_{\theta_{lv_{m}}^{\prime }}\right)\approx 0$$

(17) $$w_{m}^{\prime }\approx -\frac{1}{\rho_{0}}\frac{\partial }{\partial z}\left(\rho_{0}F_{\theta_{lv_{m}}^{\prime }}\right)/\Gamma_{\theta_{lv}}.$$

Equation 17 essentially amounts to posing the Weak Temperature Gradient (WTG) approximation (e.g., Held & Hoskins, 1985; Sobel et al., 2001), as often successfully employed in models of self‐aggregating deep convection (e.g., Ahmed & Neelin, 2019; Beucler et al., 2018; Chikira, 2014; Emanuel et al., 2014). The accuracy with which the lines in Figure 1b track each other justifies making this assumption for our shallow convective self‐aggregation too. Combining Equations 15 and 17, integrating by parts and ignoring surface flux feedbacks (which are zero by definition in our configuration with homogeneous surface fluxes) then yields a model for $\langle q_{t_{m}}^{\prime }\rangle$ which finds its energetic support solely in the heat flux anomaly $F_{\theta_{lv_{m}}^{\prime }}$, appropriately scaled by the vertical structure of the slab‐averaged, thermodynamic state:

(18) $$\frac{\partial \langle q_{t_{m}}^{\prime }\rangle }{\partial t}\approx -\langle F_{{\theta_{lv}^{\prime }}_{m}}\frac{\partial }{\partial z}\left(\frac{\Gamma_{q_{t}}}{\Gamma_{\theta_{lv}}}\right)\rangle$$

To discover why $F_{{\theta_{lv}^{\prime }}_{m}}$ develops, let us multiply fluctuations in $\theta_{lv}$ (using Equation 14) by *w*′, which decomposes the heat fluxes into flux measures of buoyancy and liquid water:

(19) $$w^{\prime }\theta_{lv}^{\prime }\equiv w^{\prime }\theta_{v}^{\prime }-7\bar{\theta_{l}}w^{\prime }q_{l}^{\prime }.$$

Figure 1c attributes the primary contribution in this decomposition to liquid water flux anomalies, that is,

(20) $$F_{{\theta_{lv}^{\prime }}_{m}}\approx -7\bar{\theta_{l}}F_{{q_{l}}_{m}}^{\prime }.$$

In turn, the divergence of $F_{{q_{l}}_{m}}^{\prime }$ stems directly from mesoscale anomalies in the condensation $C_{m}^{\prime }$. Put differently, latent heating in clouds underpins the mesoscale circulation.

Finally, as indicated in Figure 1d, convective plumes rising into a cloud layer that is moister than the slab mean will condense and later reevaporate more water vapor than average, closing a feedback loop in $q_{t_{m}}^{\prime }$. We express this feedback mathematically by assuming $F_{{q_{l}}_{m}}^{\prime }$ can be written in terms of $q_{t_{m}}^{\prime }$ through a basic mass flux approximation:

(21) $$F_{{q_{l}}_{m}}^{\prime }\approx C^{\prime }w^{*}q_{l_{m}}^{\prime }\approx Cw^{*}q_{t_{m}}^{\prime },$$

We take w* to be the root‐mean‐square vertical velocity averaged over the subcloud layer. *C* is a hypothesized model constant that subsumes the effects of (a) entrainment and detrainment from clouds, (b) considering cloud‐averaged variables rather than cloud‐core‐averaged variables and (c) conversion from $q_{l_{m}}^{\prime }$ to $q_{t_{m}}^{\prime }$.

In combination, Equations (18), (20), and 21 give a linear instability model for the moisture‐convection feedback with time scale $\tau_{q_{t_{m}}^{\prime }}$:

(22) $$\frac{\partial \langle q_{t_{m}}^{\prime }\rangle }{\partial t}\approx \frac{\langle q_{t_{m}^{\prime }}\rangle }{\tau_{q_{t_{m}}^{\prime }}},$$

(23) $$\tau_{q_{t_{m}}^{\prime }}=\frac{1}{C\bar{\theta_{l}}w^{*}\frac{\partial }{\partial z}\left(\frac{\Gamma_{q_{t}}}{\Gamma_{\theta_{lv}}}\right)}.$$

This minimal model is rather accurate for describing the evolution of $\langle q_{t_{m}}^{\prime }\rangle$ in simulation D1 (Janssens et al., 2022); here we will use it to illustrate how the mechanism is sensitive to discretization and modeling error.

## 4 Dependence on Sub‐Mesoscale Dynamics

If all assumptions made in deriving Equation 23 hold, it relies on only two variables: w* and $\partial /\partial z\left(\Gamma_{q_{t}}/\Gamma_{\theta_{lv}}\right)$. The latter of these must be positive for $\langle q_{t_{m}}^{\prime }\rangle$ to destabilize. Janssens et al. (2022) show that the required development of $\partial /\partial z\left(\Gamma_{q_{t}}/\Gamma_{\theta_{lv}}\right)$ relies only on slab‐averaged heat and moisture fluxes; so does the approximation Equation 21. Therefore, we pause for a moment to demonstrate which scales of motion control these fluxes.

Equation 20 implicitly argues that $F_{{\theta_{lv}^{\prime }}_{m}}$ is facilitated by cumulus clouds, whose energetic scales follow the depth of the boundary layer, of O(1) km. Hence, the fluctuations in vertical velocity, heat and liquid water that construct $F_{q_{l_{m}}^{\prime }}$ and $F_{\theta_{lv_{m}}^{\prime }}$ generally are of a scale much smaller than $q_{t_{m}}^{\prime }$, which by definition is larger than 12.5 km. It is therefore not trivial that $F_{\theta_{lv_{m}}^{\prime }}$ should be controlled by $q_{t_{m}}^{\prime }$ as directly as Equations 20 and 21 suggest.

**Figure 2:**
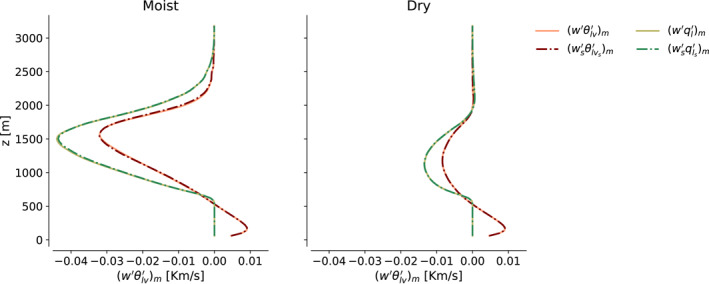
Grid‐resolved ${\left(w^{\prime }\chi^{\prime }\right)}_{m}$, for *χ* ∈ {*θ*_*lv*_, *q*_*l*_}, (*q*_*l*_ fluxes are scaled by $-7\bar{\theta_{l}}$) and pure sub‐mesoscale contributions toward this flux, ${\left(w_{s}^{\prime }\chi_{s}^{\prime }\right)}_{m}$, averaged over 10–16 hr in simulation D1, in moist (left) and dry (right) regions.

To illustrate this, consider again Figure 1d. While the mesoscale‐filtered liquid‐water flux ${\left(w^{\prime }q_{l}^{\prime }\right)}_{m}$ maps well onto the mesoscale region of high relative humidity in the upper cloud layer, the cloud structures (black contours) that carry the liquid‐water fluxes still vary as small fluctuations on top of the mesoscale moisture anomaly. As a result, almost all the convective heating underlying our mesoscale circulation is found in projections of *sub*‐mesoscale scalar fluxes onto the mesoscale. More formally, for $\chi^{\prime }\in \left\{q_{t}^{\prime },\theta_{lv}^{\prime },q_{l}^{\prime }\right\}$, one can scale‐decompose a mesoscale‐filtered vertical scalar flux as

(24) $${\left(w^{\prime }\chi^{\prime }\right)}_{m}={\left(w_{m}^{\prime }\chi_{m}^{\prime }\right)}_{m}+{\left(w_{m}^{\prime }\chi_{s}^{\prime }\right)}_{m}+{\left(w_{s}^{\prime }\chi_{m}^{\prime }\right)}_{m}+{\left(w_{s}^{\prime }\chi_{s}^{\prime }\right)}_{m}$$

and write the approximation

(25) $${\left(w^{\prime }\chi^{\prime }\right)}_{m}\approx {\left(w_{s}^{\prime }\chi_{s}^{\prime }\right)}_{m}$$

to very good accuracy, as shown for both ${\left(w^{\prime }\theta_{lv}^{\prime }\right)}_{m}$ and ${\left(w^{\prime }q_{l}^{\prime }\right)}_{m}$ in Figure 2.

Equation 25 demonstrates that a clean scale separation exists in our simulations between $w_{m}^{\prime }$ and the fluxes that produce it: In approximating the mesoscale‐filtered fluxes, one does not need to consider transport of sub‐mesoscale scalar fluctuations with the mesoscale circulation ${\left(w_{m}^{\prime }\chi_{s}^{\prime }\right)}_{m}$, dynamics contained within the mesoscale ${\left(w_{m}^{\prime }\chi_{m}^{\prime }\right)}_{m}$, or transport of mesoscale anomalies with cloudy updrafts ${\left(w_{s}^{\prime }\chi_{m}^{\prime }\right)}_{m}$. What one needs for Equation 23 to successfully explain the evolution of mesoscale moisture anomalies, is simply to correctly predict how covariability in sub‐mesoscale fluctuations of *w*, *θ*_*lv*_, and *q*_*l*_ respond to their mesoscale environment.

## 5 Sensitivity to Resolution

At Δ*x* = 200 m, our coarsest simulations barely resolve the energy‐containing scales of the shallow convection. While the impact of such assumptions may be limited in short simulations on small domains (e.g. Blossey et al., 2013; Siebesma et al., 2003), one might imagine larger sensitivities in simulations of mesoscale structures on large domains, at coarse resolutions and over long integration times.

**Figure 3:**
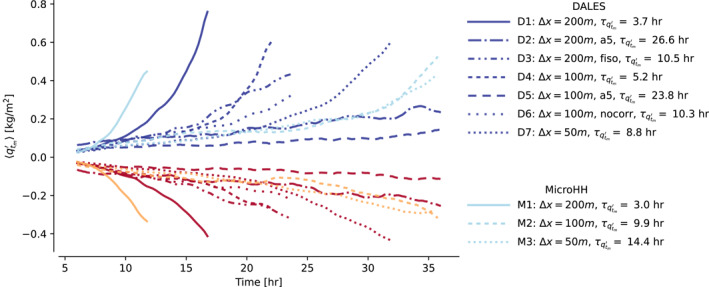
Time‐evolution of $\langle q_{t_{m}}^{\prime }\rangle$, averaged over moist (blue) and dry (red) mesoscale regions, for numerical configurations indicated by the line styles, in simulations run by the Dutch Atmospheric Large Eddy Simulaton model (dark colors) and MicroHH (light colors). Abbreviations “fiso,” “a5,” and “nocorr” follow the definitions from Table 1. Asymmetries between moist and dry regions reflect the concentration of moisture in slowly shrinking regions as self‐organization progresses.

Figure 3 presents the time evolution of vertically integrated mesoscale moisture fluctuations $\langle q_{t_{m}}^{\prime }\rangle$ for the numerical model configurations in Table 1. Each line is labeled by $\tau_{q_{t_{m}}^{\prime }}$, estimated by linear regression of Equation 22. $\tau_{q_{t_{m}}^{\prime }}$ is repeated in Table 2 along with standard errors of the fits and diagnosed model parameters of Equation 23. The results show that refining grid spacing from 200 to 50 m in the horizontal dimension more than doubles $\tau_{q_{t_{m}}^{\prime }}$ in DALES, and quadruples it in MicroHH. The models do not agree even at Δ*x* = 50 m, although they begin to drift toward each other at this resolution. If Δ*x* is kept constant, numerical setups that dissipate resolved fluctuations more strongly (simulations D2, D3, and D5) have larger $\tau_{q_{t_{m}}^{\prime }}$. In fact, switching from a second‐order advection scheme to a fifth‐order scheme (simulations D2 vs. D1 and D5 vs. D4) slows the growth of $\langle q_{t_{m}}^{\prime }\rangle$ to the point that it is barely perceptible.

**Table 2:** Results From Fitting Equations 22 and 23 to Each Simulation

	$\tau_{q_{t_{m}}}^{\prime }$ (hr)	SE (hr)	w* (m/s)	*G* (g/kg/K/m)	*C* (‐)
D1	3.70	0.19	0.557	0.00128	0.353
D2	26.6	7.26	0.539	0.00197	0.0329
D3	6.72	0.772	0.608	0.00152	0.150
D4	5.22	0.376	0.508	0.00132	0.264
D5	23.8	6.55	0.485	0.00224	0.036
D6	10.3	1.14	0.521	0.00208	0.0829
D7	8.81	0.353	0.484	0.00202	0.108
M1	2.97	0.341	0.361	0.000921	0.939
M2	9.88	0.949	0.361	0.00204	0.127
M3	14.4	1.86	0.359	0.00175	0.103

*Note*. The self‐aggregation timescale $\tau_{q_{t_{m}}}^{\prime }$ is estimated from linear regression fits of Equation 22. SE denotes the 95% confidence interval of the fits (taken to be twice the regression standard error), that is, SE does not account for sampling error in time, and should therefore be treated only as an indicator of goodness of fit. w* is obtained by averaging root‐mean‐square *w* over the subcloud layer and analysis period of each simulation. $G=\partial /\partial z\left(\Gamma_{q_{t}}/\Gamma_{\theta_{lv}}\right)$ is diagnosed in our simulations and reduced to the average over the cloud layer and analysis period. *C* is the resultant constant required to close 23.

In all numerical configurations, Equation 18 holds almost exactly (see Figures S1 and S2 in Supporting Information S1). Hence, while circulations remain responsible for driving the mesoscale moistening, and the circulations are still brought about by mesoscale heat flux anomalies acting on gradients of the mean state, either the mean state or the fluxes (or both) must react differently to a given mesoscale moisture anomaly in different numerical configurations. This is borne out in the large variations we observe in the standard errors of our linear regressions (Table 2), which indicate that a proper, linear relation does not always exist between $\langle q_{t_{m}}^{\prime }\rangle$ and $\langle F_{\theta_{lv_{m}}^{\prime }}\rangle$. This explains why some lines in Figure 3 appear to grow exponentially, while others do not. However, even when Equation 22 can be accurately fitted, we observe the model constant *C* to vary by an order of magnitude between the simulations. Since the other model parameters exhibit much less variability, this suggests that the majority of the model spread stems from how $q_{t_{m}}^{\prime }$ maps onto $F_{\theta_{lv_{m}}^{\prime }}$.

To show that this is in fact the main reason our simulations differ, we will focus on how the DALES simulations running at Δ*x* = 200 m (D1 and D3), with fifth order advection (D5) and with no stability correction (D6) differ from that running at Δ*x* = 100 m (D4). Since our length scale growth model is state‐dependent, such differences are best studied by tracing the temporal divergence between experiments that start from an identical state after the model spinup. We choose that state to be simulation D4's solution after 12 hr, when mesoscale fluctuations are small. For simulations D1 and D3, this solution is first coarse‐grained onto a grid with Δ*x* = 200 m using a top‐hat filter. We then run the cases on for 12 hr with all other settings kept identical to simulations D1, D3, D5, and D6.

**Figure 4:**
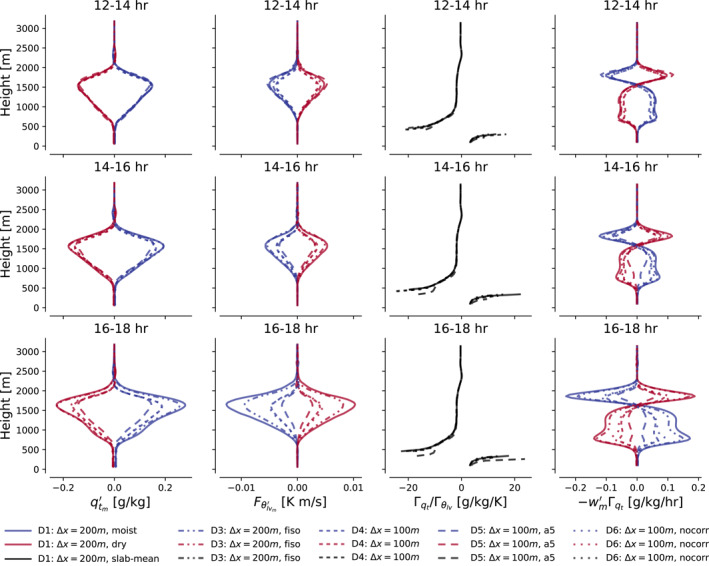
Vertical profiles of $q_{t_{m}}^{\prime }$, $F_{{\theta_{lv}^{\prime }}_{m}}$, $\Gamma_{q_{t}}/\Gamma_{\theta_{lv}}$ and $-w_{m}^{\prime }\Gamma_{q_{t}}$ (columns left to right), in moist and dry regions (blue and red lines), averaged over 2‐hr intervals (top to bottom rows) after launching the cases D1, D3, D5, and D6 from the case D4 (different line styles).

Figure 4 shows how profiles of the ingredients to Equation 18 evolve in these simulations in the first 6 hours after they have been relaunched. Their $q_{t_{m}}^{\prime }$ fields are initially identical, as is $\Gamma_{q_{t}}/\Gamma_{\theta_{lv}}$. However, this state immediately elicits a response in the coarser simulations' $F_{\theta_{lv_{m}}^{\prime }}$. It increases in strength, amplifying $w_{m}^{\prime }\Gamma_{q_{t}}$ throughout the cloud layer. As a result, $q_{t_{m}}^{\prime }$ begins growing more quickly in these simulations, supplying additional fuel that $F_{\theta_{lv_{m}}^{\prime }}$ can feed on; the feedback and divergence between the simulations intensifies over time. The main sink in the $q_{t_{m}}^{\prime }$ and $\theta_{lv_{m}}^{\prime }$ budgets, the horizontal advection term, barely responds to the changes in grid spacing (see Figures S1 and S2 in Supporting Information S1). The faster growth of $q_{t_{m}}^{\prime }$ in our coarse simulations is then not because mesoscale fluctuations are horizontally redistributed or dissipated down to the sub‐mesoscale less efficiently, or due to the WTG balance being upset. Rather, it is the enhancement of $F_{\theta_{lv_{m}}^{\prime }}$‐driven production at a given $q_{t_{m}}^{\prime }$ that accelerates the self‐organization: It is the proportionality in Equations 20 and 21 that is not grid‐converged.

**Figure 5:**
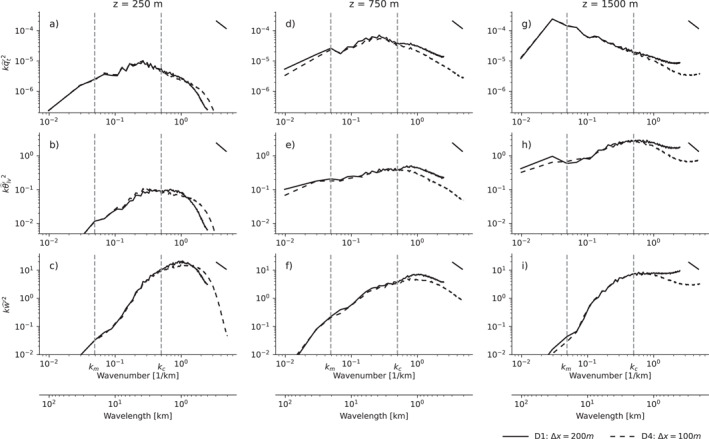
Power spectral density of *q*_*t*_ ($k{\hat{q_{t}^{\prime }}}^{2}$, a, d, g) *θ*_*lv*_ ($k{\hat{\theta_{lv}^{\prime }}}^{2}$, b, e, h) and *w* ($k{\hat{w^{\prime }}}^{2}$, c, f, i) for our Δ*x* = 100 m simulation (D4) and Δ*x* = 200 m simulation (D1) restarted from D4, averaged over the first hour after the restart, over *x*–*y* cross‐sections at 250 m (a–c, in middle of sub‐cloud layer), 750 m (d–f, in cloud layer) and 1500 m (g–i, at inversion base). *k*_*m*_ indicates the wavenumber that separates the mesoscales from the sub‐mesoscales, according to Equation 10, while *k*_*c*_ indicates the energetic length scale of the shallow convection. The top right line insets indicate *k* ^−5/3^ scaling. The spectra derive from 2D discrete Fourier transforms, whose variance is summed over radial shells and normalized to spectral density.

Why is the development of $F_{\theta_{lv_{m}}^{\prime }}$ resolution‐sensitive? The spectra plotted in Figure 5 offer a suggestion. In the first hour after the coarse‐resolution simulation D1 has been relaunched from the finer‐resolution simulation D4, it contains slightly less variance in its smallest scales of *q*_*t*_, *w*, and *θ*_*lv*_ in the sub‐cloud layer (Figures 5a–5c). But in the cloud layer, where our instability resides, fluctuations in *q*_*t*_, *w*, and *θ*_*lv*_ are more energetic at their smallest, resolved scales (Figures 5d–5f) in simulation D1 than in D4. At the inversion base, where $F_{{\theta_{lv}^{\prime }}_{m}}$ reaches its maximum, the small‐scale fluctuations in the coarse simulation are more energetic still (Figures 5g–5i).

The excess variance in cloud‐ and inversion‐layer *q*_*t*_ is initially almost ephemeral. Figure 5g shows that the inversion‐layer moisture field is dominated by its largest scales (wavenumbers smaller than *k*_*m*_), which are initially unaffected by the restart. In contrast, the variance in both *w* and *θ*_*lv*_ peaks at wavenumbers commensurate with the boundary layer height of around 2 km (marked *k*_*c*_ in Figure 5), and retains a non‐negligible contribution from a long range of scales smaller than that, especially in the cloud and inversion layers. Therefore, the excess variance in *w*′ and $\theta_{lv}^{\prime }$ at these scales might disproportionately project themselves on $F_{{\theta_{lv}^{\prime }}_{m}}$.

**Figure 6:**
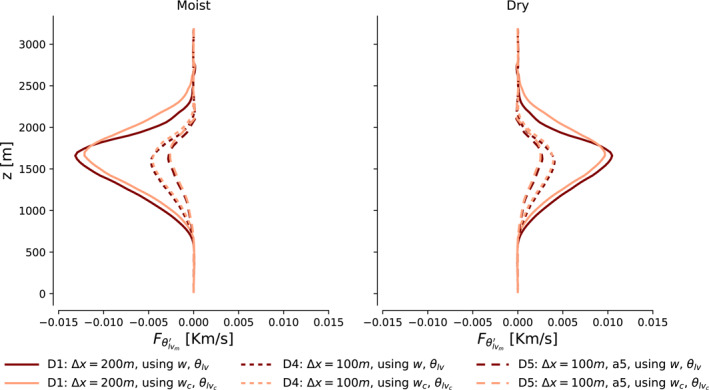
Mesoscale *θ*_*lv*_ flux anomalies (Equation 13) and their approximations using only contributions from scales smaller than 2 km (*w*_*c*_ and $\theta_{lv_{c}}$). Lines are averaged over moist and dry regions and over 16–18 hr of simulation D4 and two restarts from D4 at 12 hr: D1 and D5.

We confirm this hypothesis by evaluating the contributions toward $F_{\theta_{lv_{m}}^{\prime }}$ from length scales smaller than where the spectra begin diverging, that is, scales smaller than *k*_*c*_. Figure 6 shows that almost the entirety of $F_{\theta_{lv_{m}}^{\prime }}$ is carried by these scales (i.e., Equation 25 remains accurate even if only sub‐*k*_*c*_ scales are used), and that the resulting estimates are larger in D1 than in D4. Hence, it is the covariance of excess small‐scale *w*′ and $\theta_{lv}^{\prime }$ that underpins the stronger $F_{\theta_{lv_{m}}^{\prime }}$ in our coarse simulations at the same $q_{t_{m}^{\prime }}$, leading to a reinforced feedback.

The spectral variance plateau at the smallest, resolved scales at *z* = 1,500 m persists even when Δ*x* = 100 m, explaining why simulations D7 and M3 (Δ*x* = 50 m) self‐aggregate over an even longer time scale than simulations D4 and M2 (Δ*x* = 100 m). In fact, the plateau even persists in the inversion layer at Δ*x* = 50 m (see Figure S3 in Supporting Information S1), raising questions as to whether the self‐aggregation even in those simulations would be grid‐independent. Simulations with stronger diffusion (D3, D5, and D6) dampen the spectral plateau (see Figure S4 in Supporting Information S1), and consequently reduce $F_{{\theta_{lv}^{\prime }}_{m}}$ compared to simulation D1 (see results for D5 in Figure 6).

**Figure 7:**
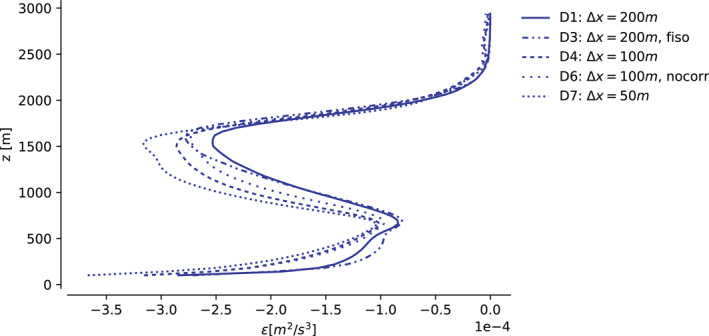
Profiles of dissipation $\varepsilon =\bar{u_{i}^{\prime }\frac{\partial \tau_{ij}^{\prime }}{\partial x_{j}}}$ of resolved turbulent kinetic energy *e*, averaged between 12 and 14 hr, for numerical configurations indicated by the line styles, in simulations run by the Dutch Atmospheric Large Eddy Simulation model. The diagnosed *ɛ* of D5 is omitted, as its dissipation cannot reliably be estimated in this manner (see e.g., Heinze et al. (2015) Section 4). All lines save D7 stem from the cases restarted from D4 at 12 hr.

So which, if any, of the results above can we trust? It is impossible to answer this question completely in the absence of observations. However, we believe we may eliminate some ambiguity by testing the degree to which the simulations hold up to the fundamental LES assumption that our quantities of interest should be independent of SFS effects. The SFS models employed in DALES and MicroHH assume these effects can reasonably be modeled by diffusion with diffusivity *K*_*m*_ ∼ *u*″*l*″, where *u*″ and *l*″ are typical velocity and length scales of the unresolved motions in the flow. This approximation can be rationalized if *l*″ ∼ Δ resides in the inertial subrange of homogeneous, isotropic turbulence. In the inertial subrange, the mean rate of transfer of turbulent kinetic energy *e* from any scale to a smaller one is scale‐independent, and equal to the rate at which it is eventually dissipated by molecular diffusion at much smaller scales, *ɛ* (e.g., Wyngaard, 2010). Therefore, we are satisfied with resolving the larger, energy‐containing eddies, characterized by velocity and length scales *U* and *L*, respectively, inserting Δ in the inertial subrange, and employing a diffusive SFS model that we only ask to model *ɛ* correctly. If it does, a necessary requirement is that *ɛ* is independent of Δ, and thus of our grid spacing (Sullivan & Patton, 2011). Figure 7 shows that this is not the case; our coarse‐mesh simulations underestimate *ɛ* with respect to our fine‐mesh simulations throughout the cloud layer, and this underdissipation accelerates the observed length scale growth (Figure S5 in Supporting Information S1 paints the same picture for our MicroHH simulations). We are either making mistakes within our model for *ɛ* at Δ*x* ∈ [100, 200] m, or must concede that these grid spacings are simply too coarse to reside in the inertial subrange.

The former is likely true for our simulations with the fifth‐order advection scheme. All our advection schemes introduce truncation errors that interact non‐trivially with the dynamics, and this makes it hard to separate numerical from modeling errors (Sullivan & Patton, 2011). The fifth‐order scheme is salient because it adds a substantial amount of diffusion to our simulation's smallest scales. If nothing is done to reduce the action of the SFS scheme, this will render the total dissipation too large, here likely resulting in such unexpected outcomes as the inhibition of shallow convective self‐aggregation at the mesoscales. To avoid having to disentangle the effects of numerical from modeled diffusion, one may co‐design one's advection and SFS schemes, for example, by letting the advection scheme's truncation error be the only diffusive source in the equations (e.g., Domaradzki et al., 2003; Hickel et al., 2006), or by casting the equations in a variational multiscale form (Hughes et al., 2000).

However, even these approaches will only work if Δ resides in the inertial subrange. Let us therefore assess some evidence that points toward this not being the case for any of our simulations. First, we address our anisotropic grid, which makes us underestimate Δ in the horizontal direction. It is in principle possible that the insufficient dissipation we observe stems from our abuse of this length scale. However, setting Δ = Δ*x* according to Deardorff (1980)'s original proposition (simulation D3) still underestimates the dissipation with respect to higher‐resolution simulations, even though it strongly overestimates the vertical component of this length scale relative to the vertical grid spacing Δ*z*. It is thus unlikely that our grid anisotropy alone is responsible for underestimating *ɛ*, though we have not assessed if this remains true at higher‐resolution combinations of Δ*x* and Δ*z*. Second, our empirical stability corrections might over‐ambitiously diminish the eddy diffusivities in stratified regions. This too could explain the excess small‐scale variance, as it rises as the stratification increases through the cloud and inversion layers. Yet, switching off the stability correction entirely (simulation D6) only slightly reduces the small‐scale variance, and does not measurably influence the evolution. Therefore, it is also unlikely that stability corrections are at the root of the problem. Third, the underestimation of dissipation is consistent across two independent LES codes with different thermodynamics and SFS models, and is thus unlikely related to individual model details.

Hence, it may be that our resolutions simply are too low to allow a proper turbulent flow to develop on the resolved scales. If we had such a flow, its large‐eddy Reynolds number Re_*L*_ ≫ 1. Following Wyngaard (1984),

(26) $${\text{Re}}_{L}=\frac{UL}{K_{m}}∼\frac{UL}{u^{\prime \prime }\Delta }∼\frac{UL}{\varepsilon^{\frac{1}{3}}\Delta^{\frac{4}{3}}}∼{\left(\frac{L}{\Delta }\right)}^{\frac{4}{3}},$$

if *ɛ* ∼ *U* ^3^/*L* ∼ *u*′′^3^/Δ, which holds if Δ resides in the inertial subrange (Tennekes & Lumley, 1972). In our simulations, *L* ∼ 1,000 m, and we attain Re_*L*_ ∼ 10 for Δ*x* ∈ [100, 200] m; this number is even lower for simulations with the O(5) advection scheme, whose effective resolution is approximately 6Δ*x* (Bryan et al., 2003). Simulations of organized, deep convection indicate that Re_*L*_ ∼ 10^2^ may be necessary for the flow to enter a regime where its statistics no longer scale with Re_*L*_ (Bryan et al., 2003); the same seems necessary for certain shallow cumulus cases (D. E. Stevens et al., 2002). Thus, grid spacings at the lower end of what we test here, or even finer, may be required to simulate organizing shallow cumulus in LES, and any subsequent transition to deep, organized convection, unless SFS models are employed that do not rely on Δ residing in the inertial subrange.

## 6 Discussion

We find that the numerical representation of fluctuations in buoyancy and vertical velocity in shallow cumuli at scales smaller than 1 km have the potential to propagate into significant differences in the moisture field at scales up to the 100 km domain sizes simulated here. We draw attention to a few implications for the modeling of tropical convection.

First, it is worthwhile to place these results in the context of early LES model intercomparisons. In the BOMEX intercomparison (Siebesma et al., 2003), small‐domain LES models agreed well with each other at the resolutions considered here. It proved much harder to achieve similar agreement for shallow cumulus under strong inversions, such as those that develop in conditions sampled during the Atlantic Tradewind Experiment (ATEX) (B. Stevens et al., 2001). It is precisely in the inversion, where the energy‐containing turbulent length scales shrink far below the boundary layer's depth (e.g., Mellado et al., 2014; Mellado et al., 2017), that we find both the key to circulation‐driven self‐aggregation, and our SFS models lacking. Given the tight coupling between the fluxes that grow the slab‐averaged cumulus layer (B. Stevens, 2007) and those that lead to its self‐aggregation (Janssens et al., 2022), we wonder whether our results simply give the historical context of the ATEX intercomparison a new perspective: It may simply be too ambitious to simulate large‐scale cloud structures that depend so strongly on hectometer‐scale plumes rising through a stratified environment at hectometer horizontal resolutions using an eddy‐viscosity SFS model.

Going further in this vein, one may question if our *vertical* grid spacing is sufficiently high to properly represent the vertical structure of the heat and moisture fluxes underlying our mechanism, especially in the transition layer that couples our subcloud and cloud layers, and the aforementioned inversion layer. Janssens et al. (2022) find the shapes of the slab‐averaged heat and moisture profiles in the transition layer to be a key ingredient for predicting the column‐integrated mesoscale moistening. Recent observations indicate that the heat and moisture fluxes through the transition layer may in nature be controlled by condensation and evaporation in a population of very shallow clouds (Albright et al., 2022). These clouds give rise to steep vertical gradients in the slab‐averaged net condensation over layers of approximately 150 m. We attempt resolve these gradients with only four vertical levels—a similar number of grid points as the effective resolution of our advection scheme. Intercomparisons of stratocumulus‐topped boundary layers indicate that transition and inversion layers remain sensitive to Δ*z* even if it is an order of magnitude finer than used here (B. Stevens et al., 2005). Since circulation‐driven moisture fluctuations in nature seem to aggregate *in* the transition layer (George et al., 2022) rather than in the inversion, as predicted by our case study and those conducted by Bretherton and Blossey (2017), Narenpitak et al. (2021), this gives ample motivation to further study of the sensitivity of mesoscale cloudiness also to vertical grid spacing in LES.

Our results also carry implications for global models that are approaching kilometer resolutions and regional models approaching hectometer resolutions. At these discretization levels, mesoscale cloud structures can be resolved. However, for example, the structures termed “flowers” by B. Stevens et al. (2020), whose development relies on the feedback Equation 18 (Narenpitak et al., 2021), remain inadequately captured in regional simulations with Δ*x* = 156 m (Schulz, 2021). Our results suggest this may be due to an overly dissipative combination of advection scheme and SFS model. Hence, another step in resolution, or parameterisations that do not require Δ to reside in the inertial scale range, may be needed for mesoscale‐resolving models to faithfully represent their mesoscale cloud structures, if they emerge from shallow convection‐driven circulations. Such parameterisations are under development for the convective “gray zone” (e.g., Honnert et al., 2020); cases of self‐organizing shallow convection should therefore pose fitting challenges to gray‐zone schemes.

At minimum, our results suggest that it is prudent for modeling studies of the spontaneous development of mesoscale shallow cloud patterns to incorporate an assessment of their degree of grid convergence. Concretely, we recommend to always assess the resolution sensitivity of one's quantities of interest, for example $\langle q_{t_{m}}^{\prime }\rangle$, and of our indicators of mesoscale variance production, for example $F_{{\theta_{lv}^{\prime }}_{m}}$ or $\tau_{q_{t_{m}}^{\prime }}$. If such sensitivities are found, inversion‐layer *w* or heat spectra may offer insight into the sensitivity's origins.

We pose our recommendations on the basis of simulations with minimal physics. Therefore, it may not be immediately obvious why our results should be of interest to situations where the mesoscales are primarily organized by radiation, precipitation or strong boundary forcings, rather than the moist convection itself. Yet, simulations of such situations often first appear to require non‐precipitating cumulus to aggregate sufficient amounts of moisture into moist mesoscale regions before developing stratiform cloud layers and cold pools (Bretherton & Blossey, 2017; Narenpitak et al., 2021), which may then modulate the mesoscale dynamics (Anurose et al., 2020; Vogel et al., 2016). Additionally, the microphysical parameterizations upon which such precipitation‐driven mechanisms rely typically exhibit even larger model biases than the turbulence parameterizations discussed here (e.g., van Zanten et al., 2011). If such parameterizations are not even driven by the right model dynamics, they can also not be expected to return realistic precipitation and cold pools. The error propagation from dynamics to physics modules for self‐organizing cumulus convection remains largely unquantified; appraising and amending such estimates is therefore a worthwhile topic of future research.

**Figure 8:**
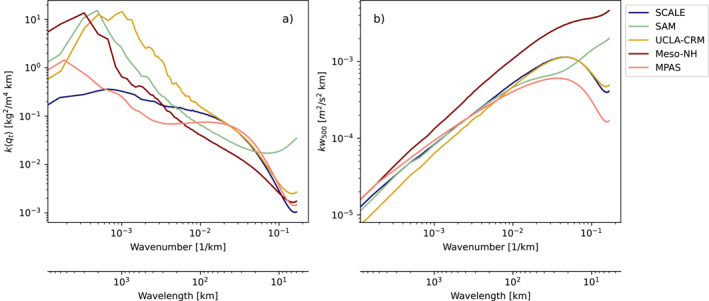
Power‐spectral densities of 〈*q*_*t*_〉 (a) and *w*_500_ (vertical velocity at 500 hPa, b) of five participating models in the Radiative‐Convective Equilibrium Model Intercomparison Project (RCEMIP), in the RCE‐large configuration detailed by Wing et al. (2018), over a sea surface at 300 K and averaged over the last 50 days of simulation. Simulations with more energetic small‐scale vertical velocity fluctuations contain more variance in their largest scales of moisture.

Finally, we return to the matter of self‐aggregation in simulations of RCE discussed in the introduction. Our coarsest two simulations (D1 and M1) develop deep convective clouds on top of their mesoscale moist regions after the period plotted in Figure 3, displaying some form of radiation‐ and precipitation‐less, deep convective self‐aggregation. We do not argue that these clouds are physical. Yet, their development does open a potential path between the convective feedback in the shallow convection discussed here and the shallow circulations that underlie deep convective self‐aggregation. Therefore, our results may help explain why numerical models set up on the same numerical domain, but with different advection schemes and SFS models, self‐aggregate so differently in RCE (Wing et al., 2020). Running with grid spacings exceeding 1 km—that is, a factor five greater than the coarsest grids used here—these simulations may simply dissipate energy from their (often parameterized) shallow convection at different rates and thus support highly variable circulation strengths and self‐aggregation time scales (Shamekh et al., 2020). The spectra of vertically integrated water vapor and vertical velocity of several simulations that participate in Wing et al. (2020) bear these hallmarks (Figure 8). More study of choices in discretization and SFS schemes, and the resulting interaction of numerical and modeling errors with the resolved dynamics in cloud‐resolving models of RCE is warranted.

## 7 Summary

In pursuit of understanding why and when idealized models of tropical convection self‐aggregate, we have studied the sensitivity to numerical settings of self‐aggregating shallow cumulus convection. In idealized LESs with a homogeneous surface forcing and no radiation or precipitation models, spontaneous aggregation is facilitated by a pure, convective instability: Small fluctuations in latent heating in shallow cumulus clouds prompt mesoscale circulations which transport moisture from dry to moist columns, resulting in aggregated cloudy patches which release more latent heat and strengthen the circulations.

The instability represents a pathway for sub‐mesoscale, turbulent fluxes of heat and moisture in kilometer‐scale cumulus clouds to control the moisture variability at scales up to two orders of magnitude larger. Therefore, modellers must take great care when trying to represent the underlying, turbulent dynamics in LES or cloud‐resolving models: We find that the time scale of the instability is highly sensitive to differences in grid spacing and advection scheme, over a range of rather conventional choices for LES modeling of shallow cumulus (Figure 3); even at Δ*x* = 50 m grid spacings, we find two LES codes with different SFS models to aggregate at rather different time scales. Given the potential role played by shallow convection in developing and maintaining deep convective self‐aggregation, we wonder whether similar differences in how cloud‐resolving models represent the effects of shallow convection matter in explaining the abundance of aggregation varieties observed in simulations of deep convection in RCE.

Our results indicate that sub‐hectometer horizontal resolution or improved SFS models may be required to adequately represent shallow convective self‐aggregation. They also call for a thorough analysis of the degree to which self‐aggregation—which slows down appreciably as our model resolution increases—matters in nature, a question which has remained elusive for studies of their deep‐convective counterparts (Muller et al., 2022). A good start in this direction is offered by simulations of the EUREC^4^A field campaign (Narenpitak et al., 2021; Saffin et al., 2022), which exhibit circulation‐driven moisture aggregation in more realistic settings, and which compare favorably to the campaign's observations. In fact, the campaign includes sufficiently detailed observations of mesoscale circulations (George et al., 2021) that the data required to reconcile models and nature may be in hand, boding well for our understanding of the role played by self‐aggregating convection in nature.

## Supporting information

Supporting Information S1

## Data Availability

Frozen images of the versions of DALES and MicroHH used in this study have been stored at https://doi.org/10.5281/zenodo.6545655 and https://doi.org/10.5281/zenodo.822842 respectively. The numerical settings, routines and post‐processed simulation data used to generate the figures presented in the manuscript are available at https://doi.org/10.5281/zenodo.7395927. Living repositories for DALES, MicroHH and the postprocessing scripts are available at https://github.com/dalesteam/dales, https://github.com/microhh/microhh and https://github.com/martinjanssens/ppagg, respectively. Both DALES and MicroHH are released under the GNU General Public License v3.0. The standardized RCEMIP data is hosted by the German Climate Computing Center (DKRZ) and is publicly available at https://www.wdc-climate.de/ui/info?site=RCEMIP_DS.
