# Supplementary material for: The Time Scale of Shallow Convective Self‐Aggregation in Large‐Eddy Simulations Is Sensitive to Numerics
Source: J Adv Model Earth Syst. 2023 Jan 4;15(1):e2022MS003292. doi: 10.1029/2022MS003292 (PMC10078365; doi:10.1029/2022MS003292)
Supplement: Supplementary file 1 — Supporting Information S1 [file JAME-15-0-s001.pdf]

Supporting Information for "The time scale of shallow convective self-aggregation in large-eddy simulations is sensitive to numerics"

Martin Janssens[1,2], Jordi Vilà-Guerau de Arellano[1], Chiel C. van Heerwaarden[1], Bart J.H. van Stratum[1], Stephan R. de Roode[2], A. Pier Siebesma[23], Franziska Glassmeier[2]

[1]Wageningen University, Wageningen, Netherlands

[2]Delft University of Technology, Delft, Netherlands

[3]KNMI, De Bilt, Netherlands

Contents of this file

1. Figures S1 to S5

Introduction

In this supplement, we present five figures that support the section "sensitivity to resolution" in the main text. Figs. S1 and S2 show how the three most important terms in the budgets for mesoscale fluctuaions of liquid-water virtual potential temperature (θ'_{lv_m}) and total water specific humidity (q'_{t_m}) are affected by changing the numerical resolution of our simulations. Figs. S3 and S4 display power spectral densities of the three most important variables underlying our simulations' self-aggregation in different numerical configurations run by MicroHH and DALES, respectively. Finally, fig. S5 indicates how dissipation of resolved turbulent kinetic energy is affected by resolution in MicroHH.

References

Bryan, G. H., Wyngaard, J. C., & Fritsch, J. M. (2003). Resolution requirements for the simulation of deep moist convection. *Monthly Weather Review*, *131*(10), 2394–2416.

de Roode, S. R., Siebesma, A. P., Jansson, F., & Janssens, M. (2022). Dependency of mesoscale organization on grid anisotropy in large-eddy simulations. *Under review at Journal of Advances in Modeling Earth Systems (JAMES).*

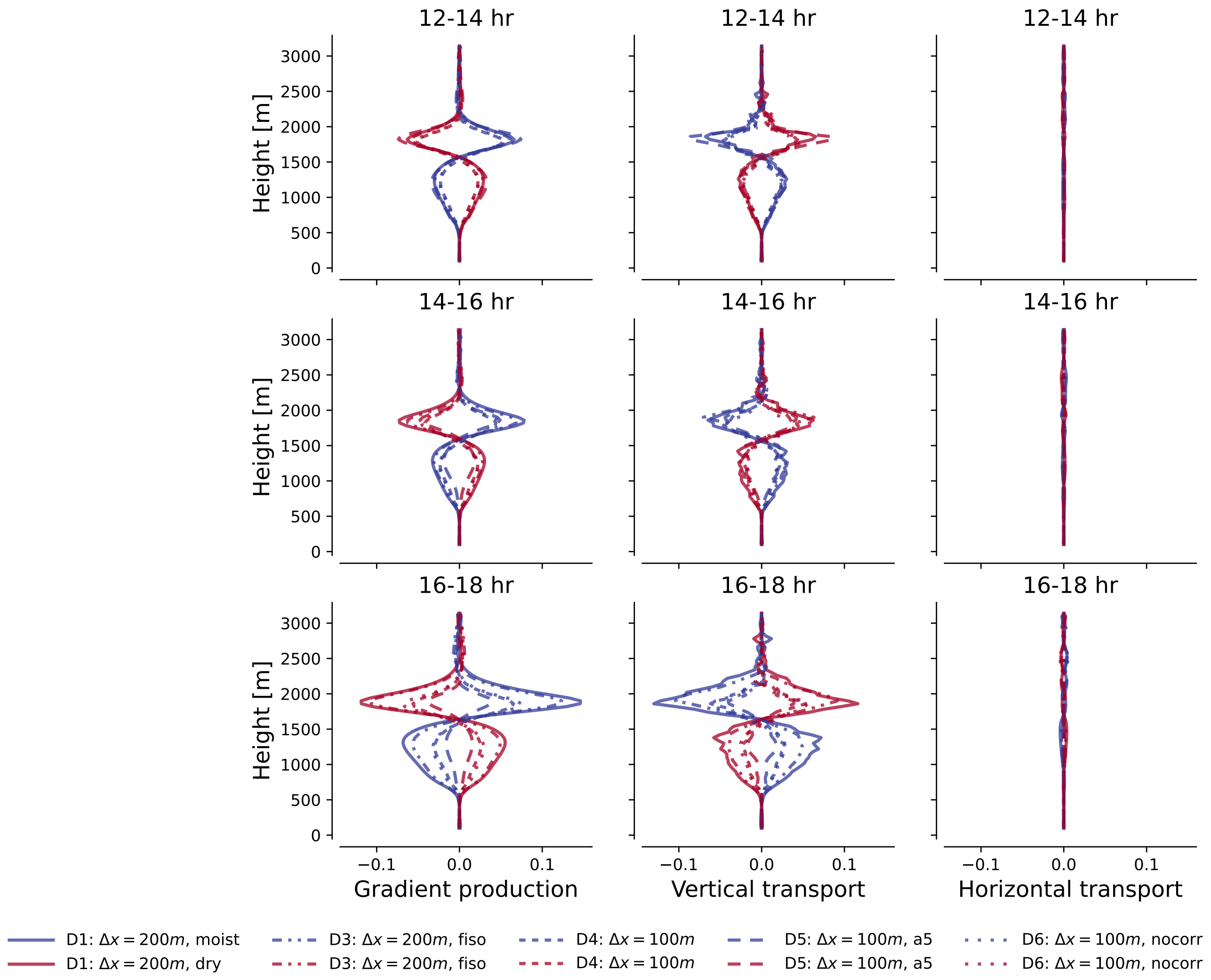

Figure S1. Vertical profiles of gradient production (left), vertical transport (centre) and horizontal transport (right) of θ'_{lv_m} evolving in time (rows) after simulations D1, D3, D5 and D6 have been launched from simulation D4 at $t = 12$ hr. All units are in K/hr. The gradient production of θ'_{lv_m} is almost exactly balanced (up to ensemble averaging deficiencies) by its vertical flux divergence, which is calculated using only sub-mesoscale contributions towards the flux following eq. 25 of the main text. Horizontal transport remains negligible. Put differently, the weak temperature gradient assumption holds well for all simulations. The upshot is that the numerical sensitivity in gradient production of q'_{t_m}, plotted in fig. S2 and discussed in the main text, can be traced to the increased vigour of the heat flux divergence in coarser simulations, plotted here.

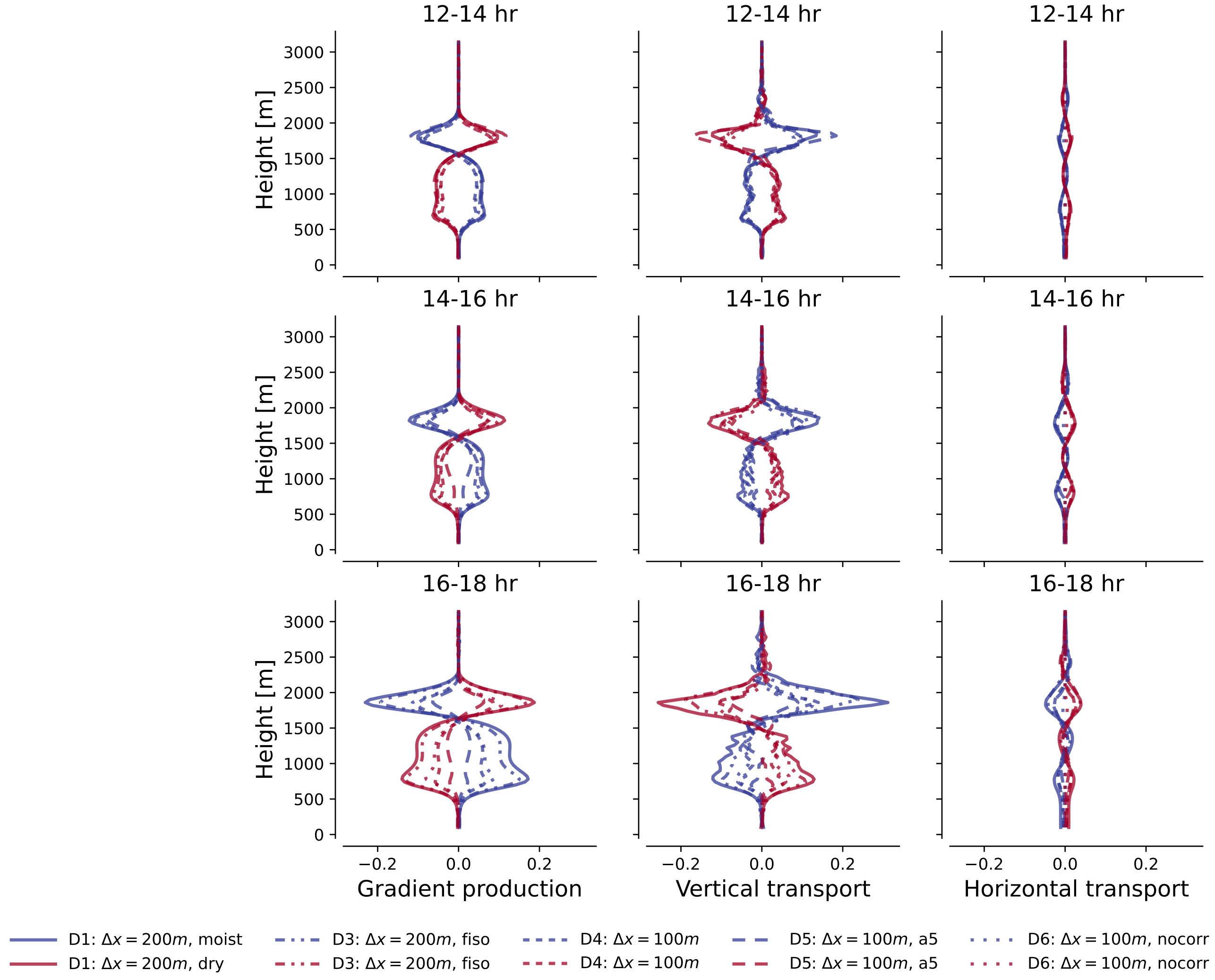

Figure S2. Vertical profiles of gradient production (left), vertical transport (centre) and horizontal transport (right) of q'_{tm} evolving in time (rows) after simulations D1, D3, D5 and D6 have been launched from simulation D4 at $t = 12$ hr. All units are g/kg/hr. Both the gradient production (which comes about through heat flux divergence, see fig. S1) and the vertical flux divergence (again calculated assuming eq. 25) intensify in coarser simulations, with exception of D5, which runs with a diffusive advection scheme that slows the growth. Horizontal moisture advection is small and unaffected by resolution change, i.e. quicker q'_{tm} growth in coarser simulations is not because they mix moisture variance horizontally and to smaller scales less efficiently, but because they produce it more efficiently.

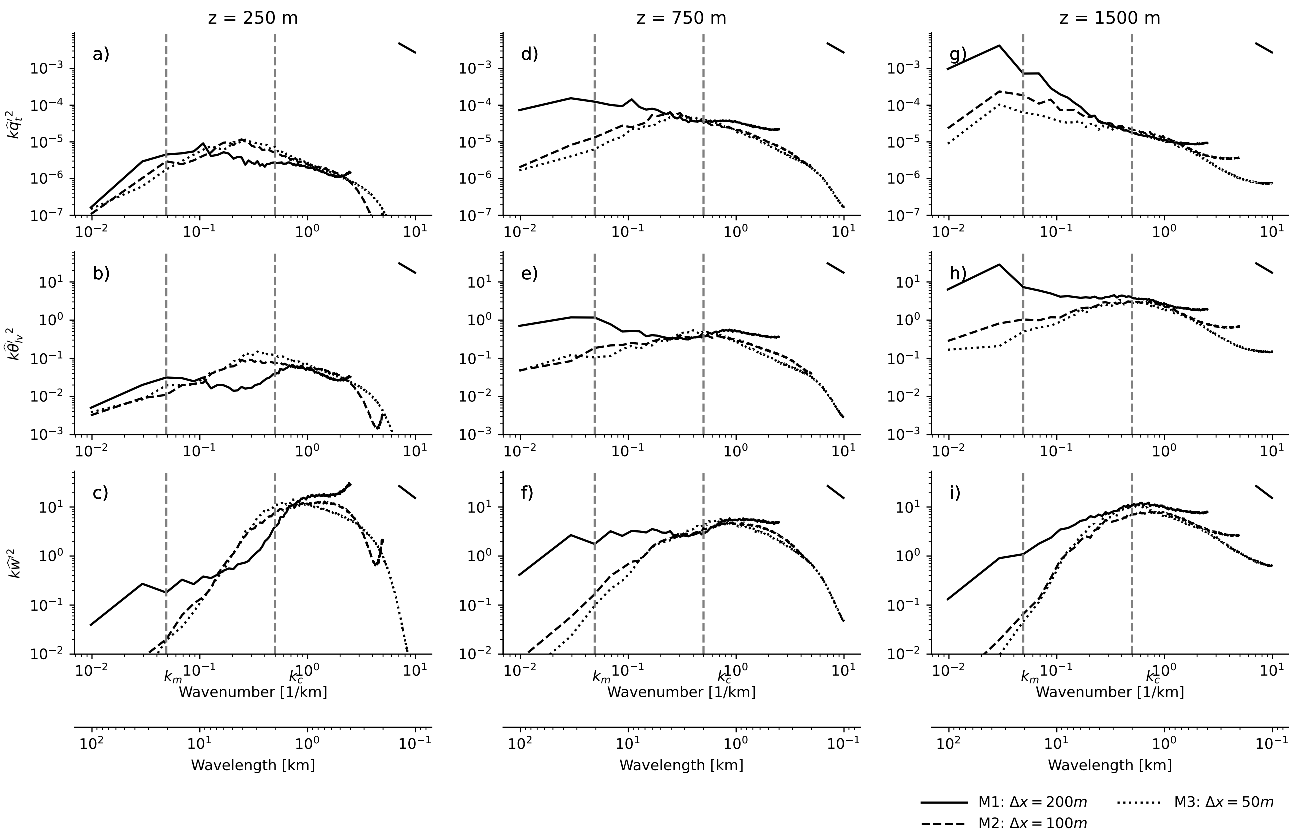

Figure S3. Radial power spectral density of q_t ($\widehat{kq_t'}^2$, a, d, g) θ_{lv} ($\widehat{k\theta_{lv}'}^2$, b, e, h) and w ($\widehat{kw'}^2$, c, f, i) for simulations M1-M3, i.e. at increasingly fine grid spacing, over x-y cross-sections at 250m (a-c, in middle of sub-cloud layer), 750m (d-f, in cloud layer) and 1500m (g-i, at inversion base). k_m indicates the wavenumber that separates the mesoscales from the sub-mesoscales. The spectra are plotted after 12 hours of simulation without restart, i.e. these spectra subsume historical information of their self-generated state, such that the excess variance predicted for the coarsest simulation (M1) is in part due to its advanced, self-reinforcing scale growth. Note, however, the same spectral variance plateaus at all three simulations' smallest, resolved scales at their inversion base, though it shifts to increasingly small, quiescent and thus inconsequential scales.

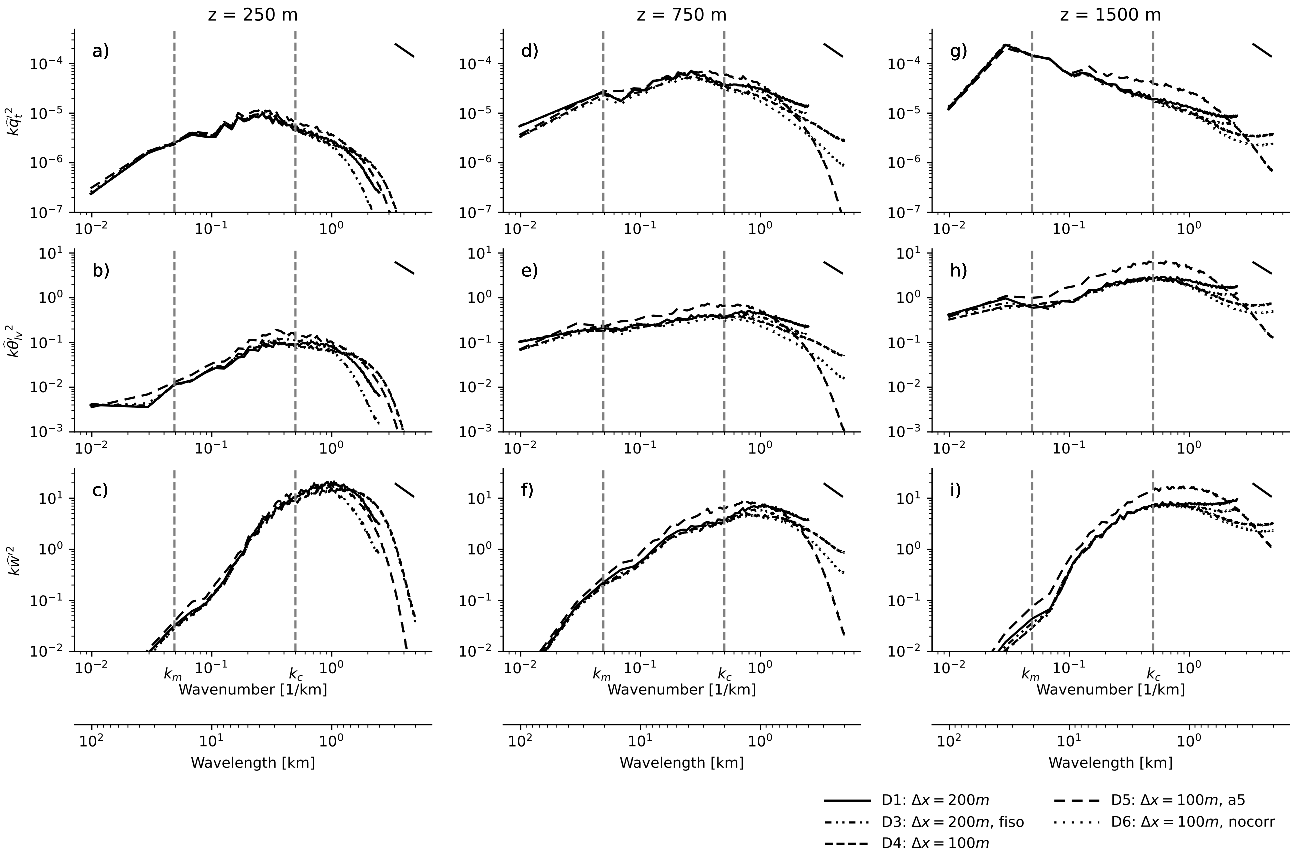

Figure S4. As fig. S3, for the three DALES simulations D1, D3 and D5 restarted from simulation D4, averaged over the first hour after the restart. The more diffusive simulations (D3 and D5) possess a reduced variance plateau at their smallest, resolved scales with respect to D1, slowing their self-aggregation. Simulation D5 appears to compensate for a lack of variability in its smallest scales - at $\Delta x = 100$m any variance < 500 m is controlled by free parameters of the numerical scheme (Bryan et al., 2003) - by shifting variance to larger scales, perhaps following the mechanism suggested by de Roode et al. (2022). Note that in spite of this, the simulation's mesoscale-filtered heat flux anomaly remains smaller than that of its 2nd order advective counterpart D4 (fig. 6 of main text). This is because almost all contributions towards this flux come from scales < 2 km, which are precisely the scales that additional numerical diffusion of D5 removes variance from.

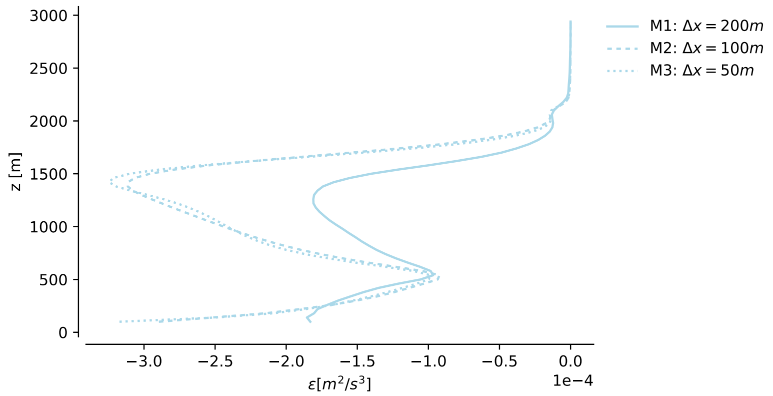

Figure S5. Profiles of dissipation ε of resolved turbulent kinetic energy e, averaged between 3-5 hr, for numerical configurations indicated by the line styles, in simulations run by MicroHH, i.e. before any of the simulations have self-aggregated appreciably. ε is much smaller in simulation M1 than in M2 and M3, consistent with this simulation being underdissipated and self-aggregating much more rapidly than its finer counterparts. Encouragingly, M2 and M3 differ less, though M2 remains underdissipated, especially at inversion base where scale growth is maximised.
